# The neuroesthetics of music as an alternative therapeutic model for enhancing youth mental wellbeing

**DOI:** 10.3389/frcha.2025.1721717

**Published:** 2026-02-24

**Authors:** K. Pradeep, Anisha Nishanth, P. Lalmohan, Lobo Manuel Alexander, S. A. Rajalakshmi

**Affiliations:** 1 Department of Visual Communication, Nehru Arts and Science College (Autonomous), Coimbatore, India; 2 Nitte Institute of Communication, Nitte (Deemed to be University), Mangaluru, Karnataka, India; 3 Department of Communication and Journalism, University of Kerala, Thiruvanathapuram, India; 4 Department of Neurology, NITTE Univarsity, Mangalore, India; 5 Department of Social Work, Amrita School of Social and Behavioural Sciences, Amrita Vishwa Vidyapeetham, Coimbatore, India

**Keywords:** neuroaesthetics, music therapy, youth mental health, cultural neuroscience, emotional regulation

## Abstract

Youth mental health challenges have escalated worldwide, particularly during adolescence and early adulthood, two critical developmental stages marked by pronounced neural plasticity, effective responsiveness, and the formation of social identity. Drawing on interdisciplinary research published in the last decade, this article critically reviews the neuroesthetics of music as an alternative and complementary therapeutic approach to promoting youth mental wellbeing. Integrating insights from neuroscience, music therapy, psychology, and cultural studies, the review examines how musical engagements activate and regulate interconnected neural systems, including the limbic, prefrontal, motor, and autonomic networks, thereby shaping reward processing, emotional regulation, stress modulation, and social bonding. Empirical studies from the past decade indicate that both passive listening and active music-making encompassing rhythm-centered interventions and movement-based practices are associated with significant reductions in anxiety and depressive symptoms, alongside improvements in attention, emotional resilience, and interpersonal synchrony. The analysis further emphasizes the cultural situatedness of musical experience, demonstrating that therapeutic outcomes are amplified when interventions are grounded in culturally embedded traditions such as Indian *raga* frameworks, Afro-Brazilian rhythmic practices, and community-oriented musical participation. In addition, the article reviews recent methodological developments, including multimodal neuroimaging, psychophysiological measures, and emerging AI-assisted adaptive sound systems, all of which enhance the precision, scalability, and personalization of music-based therapies. Taken together, this synthesis positions neuroesthetic approaches to music as a neurobiologically informed, culturally responsive, and economically accessible model with considerable potential to strengthen contemporary youth mental health interventions across diverse global contexts.

## Introduction

In an era marked by rapid social, technological, and environmental change, the mental wellbeing of youth has emerged as a global priority. Adolescence and young adulthood represent critical windows of neurodevelopment, emotional consolidation, and social identity formation. Yet, these periods are increasingly burdened by rising levels of anxiety, depression, and burnout, with one in seven adolescents worldwide affected by diagnosable mental health disorders (1, 41, 43, 44). Conventional mental health interventions, while effective, face multiple limitations, namely, limited access, cultural mismatch, stigma, and, in many regions, the scarcity of trained professionals. These restrictions highlight an urgent need for alternative, culturally resonant, and evidence-informed approaches that enhance psychological resilience, emotional regulation, and social connectedness in young populations.

Neuroesthetics, the interdisciplinary science that explores how engagement with the arts influences one’s brain, body, and behavior, creates a promising framework for addressing these challenges. At the intersection of neuroscience, psychology, and the humanities, neuroesthetics investigates the mechanisms through which esthetic understandings, ranging from music and dance to visual arts, modulate neural activity, evoke emotion, and shape cognition (2, 45). Music, in particular, has emerged as a unique potent neuroesthetic modality, which engages multiple neural networks, including the limbic, prefrontal, and motor circuits, modulating dopaminergic reward pathways and autonomic regulation (3–5). These processes translate into tangible psychosocial outcomes, namely, reductions in stress and anxiety, enhanced mood, improved attention, and strengthened social bonds (6, 46).

Unquestionably, music and dance are intrinsically human practices that are culturally embedded and socially participatory. Traditional forms of music, such as Indian *ragas*, African polyrhythms, Gregorian chants, or East Asian tonalities, combine rhythm, melody, and narrative in ways that reinforce identity, social cohesion, and emotional expression. Dance, similarly, integrates movement, memory, affect, and interpersonal synchronization, engaging mirror neuron networks and cerebellar circuits that motivate empathy, social attunement, and motor-cognitive coordination (7–9). Collectively, these embodied practices demonstrate how neuroesthetic engagement is not merely passive appreciation but an active, interactive process that influences both individual and group-level wellbeing.

Empirical evidence from global research underscores the efficiency of music interventions for mental health. Scientific studies and meta-analyses have revealed consistent benefits of dance movement therapy (DMT) for depression, social isolation, and anxiety, demonstrating improvements in mood, resilience, and interpersonal connectedness among youth and adult populations (6, 10). Music therapy, including exposure to structured or rhythm-based interventions, has been shown to reduce psychological markers of stress such as cortisol, blood pressure, and heart rate, while concurrently enhancing emotional regulation and attentional control (11–13). Neuroimaging research has revealed that anticipatory and peak emotional responses to music elicit a distinct dopamine release pattern in reward-related brain regions, offering a neurochemical explanation for music's profound affective impact (3, 4). Moreover, ethnographic, qualitative research suggests that participation in community-based music and dance programs nurtures psychosocial wellbeing by providing youth with safe spaces for identity exploration, peer support, and emotional expression (1). Simultaneously, framing music as an alternative therapeutic model represents a convergence of scientific, cultural, and clinical approaches. Unlike pharmacological or purely cognitive-behavioral approaches, music-based neuroesthetic interventions harness intrinsic human capacities for emotional resonance, pattern recognition, and embodied engagement. They are inherently low-cost, non-invasive, and adaptable across diverse sociocultural contexts, making them particularly suited for global youth populations who face barriers to conventional mental health services. Importantly, culturally informed music therapy does not replace existing interventions; instead, it complements them, enhancing adherence, engagement, and long-term efficacy by aligning with the esthetic, emotional, and social experiences of youth.

Youth mental wellbeing represents an important global challenge that demands innovative, culturally attuned, and scientifically validated approaches. Neuroesthetics, with its focus on the neural and psychosocial mechanisms of esthetic engagement, offers a compelling framework for harnessing music as an alternative therapeutic model. This research seeks to bridge existing gaps by integrating cross-cultural musical practices, mechanistic neuroscience, and practical intervention design. This article aims to provide empirical evidence supporting music's capacity to enhance emotional regulation, resilience, and social connectedness in youth, ultimately contributing to the global agenda of accessible and global mental healthcare.

## Mechanisms and therapeutic pathways of music and neuroaesthetics

The therapeutic influence of music on youth mental wellbeing arises from a dynamic interaction of neural and psychological processes. Research on neuroesthetics demonstrates that music engages multiple interconnected brain networks, generating emotional, cognitive, and social outcomes (14, 15). These neuromodulatory effects are especially significant during adolescence, a period marked by heightened plasticity in the reward, affective, and sociocognitive circuits that are highly sensitive to meaningful auditory stimuli.

Simultaneously, music produces observable shifts in autonomic functioning by modulating heart rate variability, blood pressure, and cortisol levels, all of which are core physiological indicators of stress and emotional balance (12, 13). Such psychophysiological mechanisms illustrate how musical engagement can alleviate anxiety, induce relaxation, and foster emotional stability in young populations. In addition, rhythmic and melodic patterns activate temporal and frontal cortical regions, strengthening attentional control, executive processes, and working memory domains that often undergo developmental strain during adolescence (2, 16).

Dance and embodied musical practices extend these mechanisms by integrating sensorimotor, mirror neuron, and cerebellar networks. Synchronization in dance stimulates networks implicated in empathy, social cognition, and interpersonal attunement (7, 8). These processes not only improve social connectedness but also enhance self-efficacy and agency, which are critical protective features for mental health in young people. Neuroesthetic engagement, therefore, operates at multiple levels, i.e., affective, cognitive, and social, creating a holistic framework for psychological wellbeing.

Cultural and heritage music further reinforce these pathways by shaping meaning, identity, and familiarity. For instance, Indian *ragas* organize melodic, tonal, and rhythmic elements that align with particular emotional states, thereby improving mood regulation and focus (13). Engagement with culturally meaningful music promotes identity formation and enhances social cohesion, both of which are integral to adolescence (17, 18). These results imply that music therapy informed by culture may deliver neurobiological and psychosocial support for youth wellbeing, making it applicable across different global communities.

Advanced neuroesthetic methodologies enhance both our mechanistic understanding and translational potential of music therapy. Multimodal measurement combining EEG, fMRI, and HRV allows for mapping of the brain-behavior relationship, while computational analytics quantify engagement, emotional valence, and cognitive load (19–21). These approaches facilitate personalized intervention designs, ensuring that music-based therapies can be adapted to individual neurocognitive profiles while sustaining scalability. Digital tools and AI-driven analytics further enable remote or hybrid delivery, addressing barriers such as access, stigma, and inequity, all of which are critical considerations for global youth populations.

Existing research has established the following coherent therapeutic model: musical engagement acts through neurochemical, psychophysiological, and social pathways to enhance mood, resilience, and social connectivity. For youth, this convergence of mechanisms is particularly potent, offering an accessible, culturally sensitive, and low-risk complement to conventional mental health strategies. Through elucidating the neuroesthetic pathways and integrating them with rigorous empirical measurements, researchers can develop standardized, scalable interventions capable of transforming youth mental health outcomes worldwide.

## Neuroesthetic music interventions for youth well-being

Neuroesthetic research proposes a strong scientific foundation for understanding how music operates as an alternative therapeutic approach that supports and strengthens youth mental wellbeing. Musical engagement activates a network of multisensory and emotion processing areas that collectively support reward processing, emotion regulation, and cognitive control (5, 47). When young people listen to or create music, they experience pleasurable, dopaminergic activity increases, a subsequent heightened positive affect, decreased physiological stress, and greater openness to therapeutic participation (3, 4). Evidence from adolescent and youth studies demonstrates that both active music-making, such as drumming, singing, and composing, and receptive music listening significantly reduce anxiety, enhance emotional regulation, and build resilience by lowering cortisol and improving autonomic markers such as heart rate variability (12, 13). These outcomes position music as a biologically grounded, culturally relevant, and accessible therapeutic tool that aligns naturally with youth identity and social environments, thereby strengthening its use in mental health interventions (46).

Recent empirical findings further highlight the therapeutic value of neuroesthetic principles in music-centered interventions. Randomized controlled trials and meta-analytic studies consistently report that structured music and arts interventions produce measurable enhancements in youth, alongside overall emotional stability compared to standard clinical treatments (22, 23). Active participation is especially impactful, as it nurtures interpersonal synchrony, enhances group cohesion, and reduces feelings of loneliness, factors that are particularly important for adolescents navigating emotional challenges or socioenvironmental stressors (14, 20). Moreover, rhythmic and harmonic structures in music help train the developing brain in prediction, emotional modulation, and top-down control, skills that are closely linked to long-term mental wellbeing (48, 49).

The neuroesthetic rationale for employing music as a therapeutic modality for youth must be interpreted with caution. A considerable portion of recent research is constrained by limited sample sizes, brief intervention windows, and heterogeneous study designs, which collectively weaken the robustness and generalizability of the findings (24). Moreover, the neurobiological mechanisms proposed, such as dopaminergic enhancement or strengthened emotional regulation, are frequently hypothesized rather than empirically verified in adolescent cohorts, leaving a disconnect between theoretical models and clinical outcomes (5). Variations in cultural context, musical exposure, and access further complicate their universal implementation (46). Critically, sustained therapeutic effects remain underexplored, as longitudinal and mechanistic neuroimaging studies are scarce, underscoring the need for more rigorous, developmentally attuned investigations (11). However, further longitudinal, neuroimaging, and culturally adaptable studies to deepen our mechanistic understanding and ensure broader applicability (25, 26) are required. Collectively, research reinforces music as a scientifically validated, youth-friendly, and flexible therapeutic model with exceptional potential for enhancing mental health.

## Cultural ecology of neuroesthetic music therapy and youth well-being

The cultural ecology of neuroesthetics underscores the fact that the therapeutic impact of music on youth mental wellbeing is inseparable from the social, historical, and symbolic meanings embedded within musical traditions. Across many cultures, music operates as a shared emotional grammar through which young people negotiate identity, belonging, and expressive freedom. Neuroscientific evidence shows that musical synchrony strengthens reward pathways and social bonding networks, but cultural context determines how these neurobiological effects are interpreted, valued, and sustained (27, 46). For example, in India, classical music is traditionally associated with mood-specific emotional states, offering culturally patterned mechanisms of regulation and mindfulness, while in African drumming communities, polyrhythmic participation cultivates collective resilience and intergenerational bonding (28, 29). These culturally grounded practices create predictable esthetic environments that support emotional stability, meaning-making, and youth identity formation.

However, critical perspectives reveal that health interventions often overlook these cultural subtleties. Global mental health programs frequently standardize music interventions based on Western tonal norms, which may dilute therapeutic efficacy when applied to culturally distinct populations (3, 30). Musical forms transported outside their original sociocultural contexts risk being reduced to surface-level esthetic experiences rather than carriers of communal memory, ritual significance, or spiritual function, paralleling the way classical dance loses symbolic depth when detached from its cultural setting (31). Studies from Latin America, East Asia, and the Middle East further show that youth responses to musical therapy vary significantly depending on cultural familiarity, lyrical meaning, and community values surrounding music participation (32, 33). This indicates that neuroesthetic mechanisms alone cannot explain therapeutic outcomes; rather, culturally embedded musical practices amplify neural benefits by shaping emotional interpretation, social connection, and psychological safety. Recognizing these cultural layers is essential for designing music-based mental health interventions that are ethically grounded, developmentally appropriate, and culturally resonant for diverse youth.

Moreover, movement-based music interventions such as DMT, rhythmic entrainment exercises, and structured musical physical activities demonstrate not only neurophysiological benefits but also strong cultural resonance, which amplifies their effectiveness in reducing youth stress, anxiety, and mood instability across global contexts. Evidence from controlled trials shows that rhythm-synchronized movement enhances autonomic regulation and reduces cortisol levels (10). These outcomes are shaped significantly by the cultural familiarity of the music used. School-based programs in the United States and Europe reveal that interventions grounded in locally meaningful musical genres strengthen emotional expression and peer bonding, thereby reducing internalizing symptoms (24, 34). Research from South Korea, Brazil, and India demonstrates that indigenous rhythmic traditions, such as the *samul nori*, Afro-Brazilian percussion, and folk drumming, trigger embodied emotional release and communal synchrony due to their deep cultural embeddedness (50). Meta-analyses further confirm that culturally grounded movement-music integration produces greater improvements in terms of anxiety, affect regulation, and social cohesion compared to culturally neutral interventions (14, 51). Thus, the therapeutic success of movement-based music practices is inseparable from their cultural contexts, where rhythm, identity, and collective meaning converge to support youth mental wellbeing.

## Music applications for youth mental well-being

Music engages sensory, emotional, and cognitive networks in ways that make it uniquely suited as a youth-focused mental health intervention. At the foundational level, rhythmic auditory stimulation facilitates neural entrainment, synchronizing cortical oscillations and reducing physiological stress markers. Controlled studies demonstrate significant decreases in cortisol and heart rate when young people engage with calming or rhythmically stable music, highlighting music's capacity to regulate autonomic arousal (35, 52). This foundational regulation extends into emotional domains: the amygdala ventral striatum pathway responds strongly to esthetic musical experiences, supporting emotional modulation and reducing affective volatility (5). When music takes a social form, such as group singing or drumming, it further enhances wellbeing by stimulating oxytocin release, reducing feelings of isolation, and strengthening social bonding, as shown in youth group drumming experiments (53).

These mechanisms become particularly relevant in trauma-sensitive contexts, where rhythmic grounding helps stabilize dysregulated autonomic responses. Evidence from therapeutic music studies with trauma-exposed adolescents has demonstrated reductions in post-traumatic stress disorder (PTSD) symptoms and improved emotional expression (42, 54). Beyond trauma care, music contributes to cognitive growth, with sustained musical training enhancing executive functions such as working memory, inhibition, and attention, supporting resilience in academic and social stressors (34, 55). PET neuroimaging at the reward system level confirms that pleasurable music releases dopamine in the nucleus accumbens and increases motivation, an essential factor for youth experiencing anhedonia or disengagement (56).

Music enhances vagal tone when paired with controlled breathing or slow tempos, thereby reducing anxiety and supporting emotion-focused coping (36, 57). When combined with movement, as in DMT, music promoted somatic emotional release and decreased internalizing symptoms in multiple clinical trials (37, 38). Global studies provide robust evidence for the application of music in youth mental health. In Europe and North America, school-based music interventions have been shown to reduce anxiety, depressive symptoms, and behavioral dysregulation among adolescents (1, 58). Programs integrating music therapy, group dance, and rhythmic improvisation have been associated with improved attention, emotional regulation, and interpersonal skills (10, 59). Social prescribing initiatives, particularly in the United Kingdom, have formally recognized music engagement as a non-pharmacological intervention capable of enhancing psychosocial wellbeing and reducing reliance on medicalized approaches (35, 39).

In a developing country like India, the cultural resonance of music provides a compelling platform for neuroesthetic interventions. Indian classical music, particularly *raga* therapy, has demonstrated measurable effects on automatic regulation, anxiety reduction, and mood enhancement (12, 17). Pilot studies in Indian educational and community settings indicate that music-based interventions improve concentration, self-regulation, and social connectedness, suggesting pathways for scalable youth mental health programs (21, 40). Further, the horizon of AI-driven adaptive soundscapes offers a scalable neuroesthetic tool, with recent trials demonstrating measurable reductions in daily stress among young adults. Thus, neuroesthetic music interventions operate through physiological, social, cognitive, emotional, and cultural pathways, forming a robust therapeutic model for enhancing youth mental wellbeing.

## Future directions for neuroesthetic music interventions

Future research on neuroesthetic music interventions for youth mental health must pursue more methodologically rich, culturally grounded, and mechanistically explicit research pathways to deepen both theoretical and clinical insights. Longitudinal neuroimaging designs are particularly essential for mapping how music-based therapeutic programs progressively modify neural networks implicated in reward processing, affective regulation, and executive functioning across distinct developmental periods. The evidence is critical for assessing whether short-term improvements extend into enduring neuroplastic adaptations. Equally important is the development of culturally responsive intervention frameworks that integrate indigenous musical forms, contextual meaning, and community-based practices. Bringing this about requires participatory collaboration with young people, educators, and cultural knowledge bearers. Furthermore, future investigations must disentangle music-specific mechanisms through careful control of confounding influences, such as physical movement, novelty, or social engagement, within multimodal interventions. Large-scale randomized controlled trials conducted in schools, community settings, and digital ecosystems will further enhance ecological validity and capture the heterogeneity of youth populations. Emerging innovations, including AI-driven adaptive sound environments and biofeedback-integrated music platforms, present compelling opportunities but necessitate transparent algorithmic design, developmental appropriateness, and rigorous cross-cultural evaluation. Collectively, advancing these research trajectories will solidify neuroesthetic music therapy as a scientifically credible, contextually inclusive, and globally scalable model for promoting youth mental wellbeing.

## Conclusion

The study demonstrates that neuroesthetic engagement with music constitutes a strong, evidence-based therapeutic avenue for advancing youth mental wellbeing. Integrating research from neuroscience, psychology, music therapy, and cultural studies, musical engagement consistently modulates limbic prefrontal, sensorimotor, and autonomic neural networks, supporting stress reduction, emotional regulation, attentional control, reward processing, and social connectedness during adolescence and young adulthood, developmental phases characterized by heightened neuroplasticity and psychosocial vulnerability. These neurobiological mechanisms are reinforced through psychophysiological stabilization and social processes, predominantly within participatory and embodied modalities such as music-making and dance-based interventions that activate interpersonal synchrony, empathy, and agency. Crucially, this study highlights the fact that therapeutic efficacy is not exclusively driven by neural mechanisms but is shaped significantly by cultural embeddedness. Music-based interventions grounded in culturally familiar traditions, rhythm-based practices, and community rhythmic forms enhance identity coherence, engagement, and emotional safety, thereby amplifying psychosocial outcomes. Despite neuroesthetic music-centered therapy being a low-cost, non-invasive, and scalable complement to conventional mental health approaches, limitations persist due to methodological heterogeneity and a lack of longitudinal, mechanistic neuroimaging studies in youth populations. Addressing these gaps through culturally attuned, developmentally sensitive, and multimodal research designs is essential. Overall, neuroesthetic music therapy emerges as a credible, integrative framework with significant potential for application across educational, community, and public health contexts to advance global youth mental health.

## Data Availability

The original contributions presented in the study are included in the article/Supplementary Material, further inquiries can be directed to the corresponding authors.
